# Supplementary material for: Taking a snapshot of the triplet excited state of an OLED organometallic luminophore using X-rays
Source: Nat Commun. 2020 May 1;11:2131. doi: 10.1038/s41467-020-15998-z (PMC7195477; doi:10.1038/s41467-020-15998-z)
Supplement: Supplementary file 1 — Supplementary Information [file 41467_2020_15998_MOESM1_ESM.pdf]

Supplementary Information

Taking a snapshot of the Triplet Excited State of an OLED Organometallic Luminophore using X-rays

Smolentsev et al.

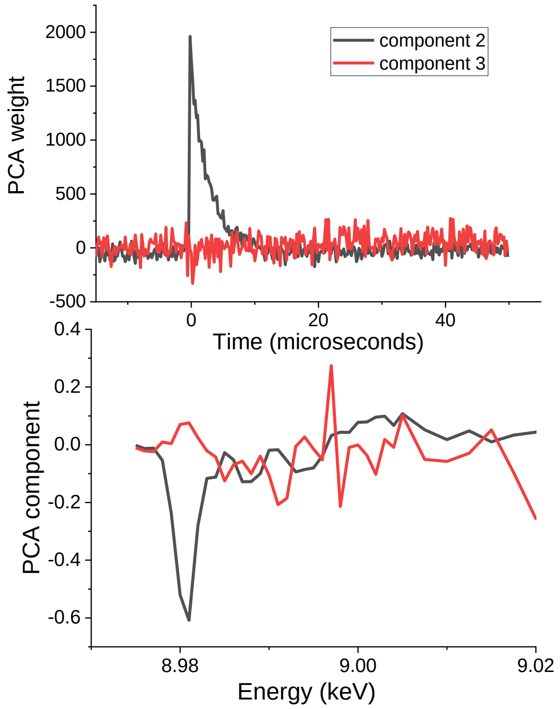

Supplementary Figure 1. Weights of components 2 and 3 extracted using principal component analysis from the time series of XAS spectra (top) and corresponding spectral components (bottom). Component 1 is almost identical to the averaged XAS spectrum and is not shown, component 2 represents transient XAS signal and was fully analyzed, while component 3 is dominated by the statistical noise.

Supplementary Methods

In order to estimate the average charge change at P atoms we have assumed a linear relationship between the shift of XES δE and charge changes at P atoms δq: $\delta E = A\delta q$ The validity of this approximation for XES spectra of P has been demonstrated in the literature[1,2]. Since the shift is very small it cannot be measured by comparing the position of maxima for laser-on and laser-off spectra. Instead one can use the expansion of spectrum as a function of energy with one order of smallness: $\mathrm{XES}(E, \delta E) = \mathrm{XES}(E) + \frac{d\mathrm{XES}}{dE}(E)\delta E$. Then the difference between excited and ground state spectra for a charge change δq can be calculated as $\Delta\mathrm{XES}(E, \delta q) = \frac{d\mathrm{XES}}{dE}(E)A\delta q$ The transient signal scales down with the excited state fraction α : $\mathrm{TransientXES}(E, \delta q) = \alpha\frac{d\mathrm{XES}}{dE}(E)A\delta q$. Coefficient A is known from the measurements for reference compounds[1,2]. It depends on which charge is used for calibration: formal oxidation state or atomic charge calculated using DFT. Both calibrations were taken from literature[1,2]. $\mathrm{TransientXES}(E)$ and $\mathrm{XES}(E)$ we have measured experimentally. Therefore, by fixing high estimate for the excited state fraction α the low estimate for the charge change δq can be obtained.

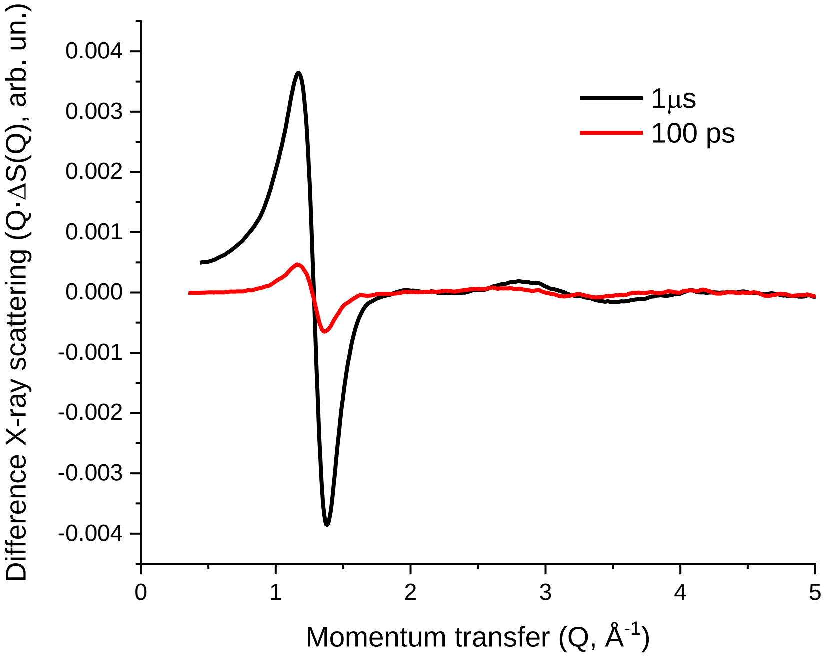

Supplementary Figure 2. Pump-probe X-ray scattering signals after ultrafast heating of THF solvent, corresponding to the temperature change (measured at 100 ps delay) and density change (measured at 1 μs delay).

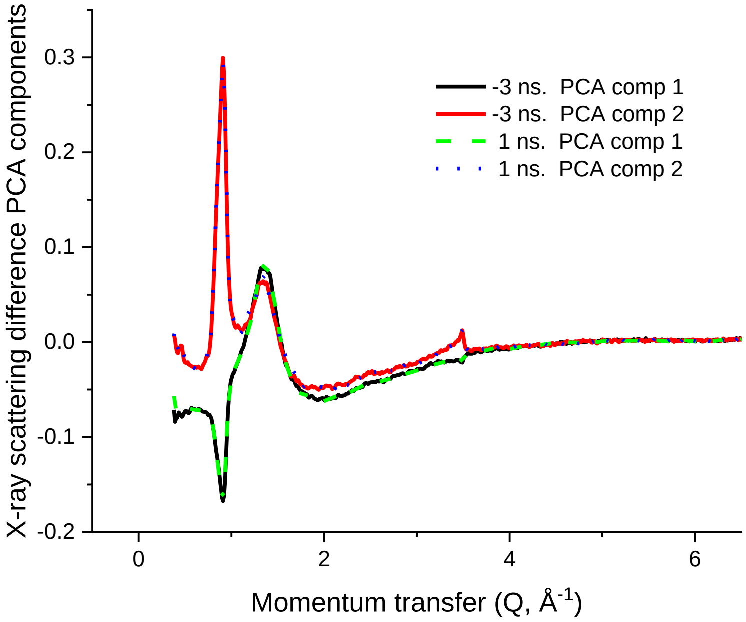

Supplementary Figure 3. Two components identified in the individual pump-probe X-ray scattering patterns, measured for delays -3 ns and 1 ns. Components were extracted using principal component analysis and represent fluctuations of the background.

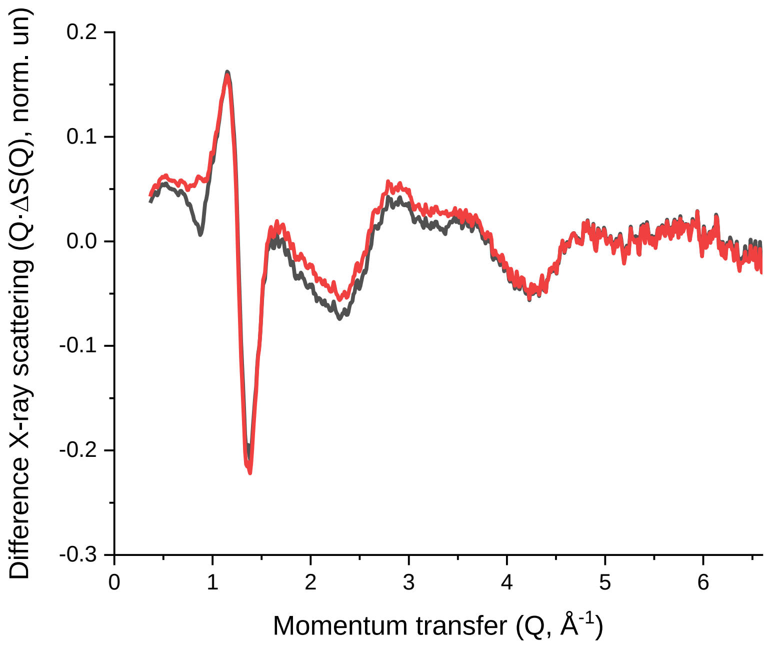

Supplementary Figure 4. Pump-probe X-ray scattering signals for delay time 1ns which were obtained by averaging of individual X-ray scattering patterns (black line) and after removal of the PCA-identified background components (red line).

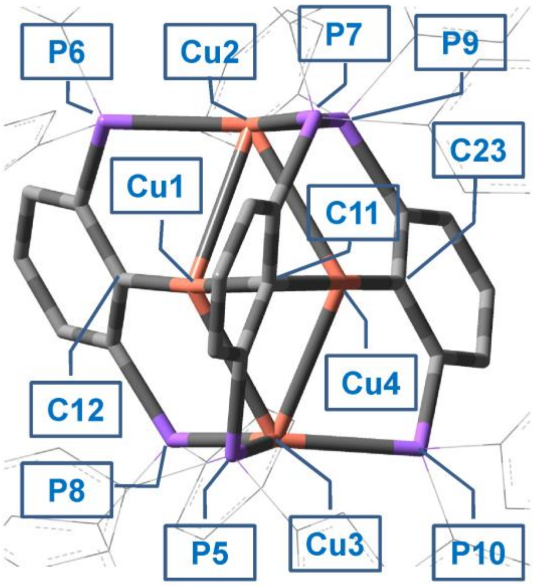

Supplementary Figure 5. Labeling of atoms of $[Cu_4(PCP)_3]^+$ used in Tables S1 and S2.

Supplementary Table 1. Atomic charges q (a.u.) in the S_0 / T_1 state of the $[Cu_4(PCP)_3]^+$ ion calculated with Bader and Mulliken approaches with the ADF-2018 package. The charge variations $\Delta q = q(T_1) - q(S_0)$ are given

Mulliken charge analysis											
**DFT level**	**State**	**Cu1**	**Cu2**	**Cu3**	**Cu4**	**P5**	**P8**	**P10**	**P6**	**P7**	**P9**
B3LYP/ DZP	S_0	0.331	-0.011	-0.004	0.300	0.844	0.828	0.833	0.826	0.826	0.840
	T_1	0.420	-0.028	-0.031	0.362	0.858	0.827	0.845	0.813	0.817	0.844
	Δq	0.088	-0.017	-0.027	0.061	0.013	-0.000	0.012	-0.013	-0.009	0.003
B3LYP/ TZ2P	S_0	-0.030	-0.261	-0.252	0.038	0.830	0.779	0.742	0.731	0.758	0.825
	T_1	-0.020	-0.278	-0.288	0.050	0.829	0.775	0.754	0.725	0.73	0.813
	Δq	0.009	-0.017	-0.036	0.011	-0.001	-0.004	0.011	-0.005	-0.028	-0.012
B3LYP/ QZ4P	S_0	0.072	0.840	0.685	0.345	0.952	1.046	0.923	0.944	0.939	1.006
	T_1	0.124	0.881	0.800	0.427	0.896	1.036	0.939	0.890	0.945	1.004
	Δq	0.052	0.041	0.115	0.082	-0.056	-0.01	0.016	-0.053	0.005	-0.002
TPSS/ TZ2P	S_0	-0.066	-0.414	-0.384	0.027	0.823	0.793	0.758	0.746	0.755	0.857
	T_1	-0.079	-0.440	-0.432	0.027	0.840	0.792	0.780	0.752	0.736	0.853
	Δq	-0.013	-0.026	-0.047	-0.000	0.017	-0.001	0.022	0.006	-0.018	-0.004
B3LYP*/ TZ2P	S_0	-0.038	-0.292	-0.280	0.030	0.838	0.788	0.751	0.740	0.767	0.834
	T_1	-0.032	-0.309	-0.317	0.039	0.839	0.784	0.766	0.736	0.739	0.823
	Δq	0.005	-0.016	-0.037	0.009	0.001	-0.004	0.015	-0.004	-0.027	-0.011
TPSSH/ TZ2P	S_0	-0.045	-0.342	-0.317	0.044	0.800	0.768	0.737	0.725	0.733	0.833
	T_1	-0.052	-0.369	-0.361	0.047	0.817	0.768	0.754	0.727	0.714	0.827
	Δq	-0.006	-0.027	-0.043	0.002	0.016	-0.000	0.016	0.002	-0.019	-0.005
GGA-PBE/ TZ2P	S_0	-0.052	-0.400	-0.385	0.021	0.911	0.871	0.829	0.818	0.841	0.923
	T_1	-0.062	-0.417	-0.427	0.018	0.924	0.869	0.85	0.819	0.823	0.915
	Δq	-0.009	-0.016	-0.042	-0.002	0.013	-0.002	0.021	0.001	-0.018	-0.007
TPSS/ QZ4P	S_0	-0.652	0.344	0.313	-0.623	0.535	0.654	0.619	0.670	0.642	0.673
	T_1	-0.804	0.459	0.396	-0.603	0.593	0.631	0.668	0.656	0.639	0.667
	Δq	-0.152	0.114	0.083	0.019	0.057	-0.023	0.048	-0.014	-0.002	-0.005
CAMY-B3LYP/ QZ4P	S_0	0.070	0.764	0.649	0.258	0.802	0.902	0.824	0.829	0.803	0.881
	T_1	0.113	0.807	0.740	0.370	0.776	0.892	0.829	0.790	0.814	0.879
	Δq	0.043	0.043	0.092	0.112	-0.025	-0.010	0.005	-0.040	0.011	-0.001
**Bader charge analysis**											
**Vxc**	**Spin**	**Cu1**	**Cu2**	**Cu3**	**Cu4**	**P5**	**P8**	**P10**	**P6**	**P7**	**P9**
B3LYP/ DZP	S_0	0.333	0.252	0.266	0.337	1.219	1.227	1.226	1.231	1.238	1.220
	T_1	0.419	0.228	0.249	0.457	1.289	1.257	1.235	1.218	1.225	1.243
	Δq	0.085	-0.024	-0.017	0.119	0.069	0.030	0.009	-0.013	-0.012	0.022
B3LYP/ TZ2P	S_0	0.349	0.260	0.274	0.355	1.305	1.320	1.313	1.327	1.332	1.323
	T_1	0.433	0.236	0.255	0.466	1.369	1.339	1.330	1.302	1.304	1.336
	Δq	0.084	-0.023	-0.019	0.110	0.064	0.019	0.016	-0.024	-0.028	0.012
B3LYP/ QZ4P	S_0	0.352	0.268	0.273	0.357	1.490	1.509	1.491	1.509	1.502	1.497
	T_1	0.456	0.224	0.263	0.461	1.532	1.531	1.518	1.482	1.496	1.518
	Δq	0.103	-0.043	-0.009	0.103	0.042	0.022	0.026	-0.027	-0.006	0.021
TPSS/ TZ2P	S_0	0.362	0.279	0.292	0.369	1.318	1.326	1.309	1.337	1.338	1.325
	T_1	0.418	0.265	0.280	0.434	1.376	1.342	1.340	1.307	1.316	1.347

	Δq	0.055	-0.013	-0.011	0.065	0.058	0.016	0.030	-0.030	-0.021	0.021
B3LYP*/ TZ2P	S_0	0.347	0.249	0.264	0.344	1.281	1.284	1.263	1.293	1.297	1.289
	T_1	0.411	0.228	0.246	0.445	1.354	1.304	1.303	1.277	1.281	1.310
	Δq	0.064	-0.021	-0.017	0.100	0.072	0.019	0.039	-0.015	-0.016	0.021
TPSSH/ TZ2P	S_0	0.367	0.302	0.295	0.372	1.366	1.387	1.392	1.389	1.379	1.376
	T_1	0.452	0.260	0.292	0.458	1.428	1.399	1.400	1.372	1.387	1.401
	Δq	0.085	-0.042	-0.002	0.085	0.061	0.012	0.008	-0.017	0.007	0.024
GGA-PBE/ TZ2P	S_0	0.348	0.254	0.265	0.357	1.223	1.238	1.224	1.233	1.235	1.239
	T_1	0.405	0.236	0.253	0.418	1.304	1.256	1.25	1.231	1.238	1.259
	Δq	0.056	-0.018	-0.011	0.061	0.081	0.018	0.025	-0.002	0.003	0.020
TPSS/ QZ4P	S_0	0.365	0.279	0.291	0.368	1.498	1.502	1.484	1.506	1.509	1.503
	T_1	0.443	0.245	0.292	0.424	1.536	1.517	1.533	1.483	1.513	1.526
	Δq	0.078	-0.034	0.000	0.056	0.038	0.014	0.048	-0.023	0.003	0.022
CAMY-B3LYP/ QZ4P	S_0	0.357	0.280	0.285	0.363	1.513	1.527	1.517	1.520	1.526	1.524
	T_1	0.470	0.233	0.273	0.498	1.558	1.543	1.538	1.504	1.520	1.546
	Δq	0.113	-0.047	-0.013	0.135	0.045	0.017	0.021	-0.016	-0.006	0.022

Supplementary Table 2. Atomic charges q (a.u.) in the S_0 / T_1 state of the $[Cu_4(PCP)_3]^+$ ion calculated with Bader, NBO and Mulliken approaches with the Gaussian 09 package. The charge variations Δq = q(T_1) - q(S_0) are given.

**Mulliken charge analysis**											
**DFT level**	**State**	**Cu1**	**Cu2**	**Cu3**	**Cu4**	**P5**	**P8**	**P10**	**P6**	**P7**	**P9**
B3LYP/ DGDZVP	S_0	-0.449	-0.412	-0.399	-0.442	0.754	0.695	0.708	0.659	0.696	0.727
	T_1	-0.402	-0.428	-0.434	-0.474	0.777	0.702	0.709	0.646	0.669	0.725
	Δq	0.047	-0.016	-0.035	-0.032	0.023	0.007	0.001	-0.013	-0.027	-0.002
B3LYP/ 6-311 G(d,p)	S_0	0.701	0.773	0.709	0.819	0.275	0.256	0.312	0.251	0.237	0.254
	T_1	0.955	0.707	0.683	0.895	0.229	0.273	0.286	0.209	0.248	0.249
	Δq	0.254	-0.066	-0.026	0.076	-0.046	0.017	-0.026	-0.042	0.011	-0.005
M06/ DGDZVP	S_0	-0.451	-0.289	-0.292	-0.446	0.709	0.628	0.666	0.608	0.642	0.649
	T_1	-0.369	-0.329	-0.324	-0.466	0.708	0.623	0.651	0.585	0.623	0.637
	Δq	0.082	-0.040	-0.032	-0.020	-0.001	-0.005	-0.015	-0.023	-0.019	-0.012
**Bader charge analysis**											
B3LYP/ DGDZVP	S_0	0.328	0.227	0.232	0.332	1.507	1.516	1.509	1.517	1.514	1.496
	T_1	0.393	0.190	0.223	0.399	1.549	1.529	1.541	1.514	1.515	1.535
	Δq	0.065	-0.037	-0.009	0.067	0.042	0.013	0.032	-0.003	0.001	0.039
B3LYP/ 6-311 G(d,p)	S_0	0.417	0.380	0.383	0.422	1.519	1.526	1.524	1.531	1.528	1.522
	T_1	0.534	0.362	0.376	0.536	1.553	1.538	1.553	1.521	1.526	1.540
	Δq	0.117	-0.018	-0.007	0.114	0.034	0.012	0.029	-0.010	-0.002	0.018
M06/ DGDZVP	S_0	0.303	0.205	0.210	0.308	1.498	1.506	1.499	1.508	1.504	1.504
	T_1	0.375	0.165	0.197	0.366	1.537	1.519	1.532	1.505	1.505	1.524
	Δq	0.072	-0.040	-0.013	0.058	0.039	0.013	0.033	-0.003	0.001	0.020
**NBO charge analysis**											
B3LYP/	S_0	0.500	0.115	0.125	0.505	0.902	0.910	0.902	0.903	0.898	0.913

DGDZVP	T_1	0.600	0.029	0.114	0.564	0.917	0.914	0.928	0.909	0.907	0.932
	Δq	0.100	-0.086	-0.011	0.059	0.015	0.004	0.026	0.006	0.009	0.019
B3LYP/ 6-311 G(d,p)	S_0	0.749	0.821	0.819	0.753	0.741	0.761	0.756	0.752	0.739	0.759
	T_1	0.970	0.812	0.824	0.938	0.742	0.760	0.765	0.722	0.739	0.759
	Δq	0.221	-0.009	0.005	0.185	0.001	-0.001	0.009	-0.030	0	0
M06/ DGDZVP	S_0	0.478	0.097	0.106	0.488	0.918	0.926	0.918	0.918	0.913	0.928
	T_1	0.578	0.003	0.089	0.530	0.935	0.929	0.945	0.923	0.920	0.946
	Δq	0.100	-0.094	-0.017	0.042	0.017	0.003	0.027	0.005	0.007	0.018

Supplementary Table 3. Differences between average charges of P-coordinated Cu, C-coordinated Cu and P atoms for excited triplet and ground state singlet states. Used functionals are B3LYP (B3LYP*)[3], TPSS (TPSSh)[4], GGA-PBE[5], CAMY-B3LYP[6], and M06[7].

Code	Level of theory	Charge analysis method	Average change of charges (triplet-singlet)		
			Cu(P-coord)	Cu (C-coord)	P
ADF	B3LYP/ DZP	Mulliken	-0.022	0.075	0.001
ADF	B3LYP/ TZ2P	Mulliken	-0.027	0.011	-0.007
ADF	B3LYP/ QZ4P	Mulliken	0.078	0.067	-0.017
ADF	TPSS/ TZ2P	Mulliken	-0.037	-0.007	0.004
ADF	B3LYP*/ TZ2P	Mulliken	-0.003	0.007	-0.005
ADF	TPSSh/ TZ2P	Mulliken	-0.036	-0.002	0.001
ADF	GGA-PBE/ TZ2P	Mulliken	-0.029	-0.006	0.001
ADF	TPSS/ QZ4P	Mulliken	0.099	-0.066	0.010
ADF	CAMY-B3LYP/ QZ4P	Mulliken	0.067	0.077	-0.010
ADF	B3LYP/ DZP	Bader	-0.021	0.103	0.018
ADF	B3LYP/ TZ2P	Bader	-0.021	0.097	0.010
ADF	B3LYP/ QZ4P	Bader	-0.027	0.104	0.013
ADF	TPSS/ TZ2P	Bader	-0.013	0.061	0.010
ADF	B3LYP*/ TZ2P	Bader	-0.019	0.082	0.020
ADF	TPSSh/ TZ2P	Bader	-0.023	0.085	0.016
ADF	GGA-PBE/ TZ2P	Bader	-0.015	0.059	0.024
ADF	TPSS/ QZ4P	Bader	-0.017	0.067	0.017
ADF	CAMY-B3LYP/ QZ4P	Bader	-0.030	0.124	0.014
Gaussian	B3LYP/ DGDZVP	Mulliken	-0.026	0.008	-0.002
Gaussian	B3LYP/6311G(d,p)	Mulliken	-0.046	0.165	-0.015
Gaussian	M06/ DGDZVP	Mulliken	-0.036	0.031	-0.013
Gaussian	B3LYP/ DGDZVP	Bader	-0.023	0.066	0.021
Gaussian	B3LYP/ 6-311G(d,p)	Bader	-0.013	0.116	0.014
Gaussian	M06/DGDZVP	Bader	-0.027	0.065	0.017
Gaussian	B3LYP/DGDZVP	NBO	-0.049	0.080	0.013
Gaussian	B3LYP /6311G(d,p)	NBO	-0.002	0.203	-0.004
Gaussian	M06/ DGDZVP	NBO	-0.056	0.071	0.013

Supplementary References

1. Petric, M. & Kavčič, M. Chemical speciation via X-ray emission spectroscopy in the tender X-ray range. *J. Anal. At. Spectrom.* **31**, 450–457 (2016).
2. Petric, M. *et al.* Chemical State Analysis of Phosphorus Performed by X-ray Emission Spectroscopy. *Anal. Chem.* **87**, 5632–5639 (2015).
3. Reiher, M., Salomon, O. & Artur Hess, B. Reparameterization of hybrid functionals based on energy differences of states of different multiplicity. *Theor Chem Acc* **107**, 48–55 (2001).
4. Tao, J., Perdew, J. P., Staroverov, V. N. & Scuseria, G. E. Climbing the Density Functional Ladder: Nonempirical Meta--Generalized Gradient Approximation Designed for Molecules and Solids. *Phys. Rev. Lett.* **91**, 146401 (2003).
5. Perdew, J. P., Burke, K. & Ernzerhof, M. Generalized Gradient Approximation Made Simple. *Phys. Rev. Lett.* **77**, 3865–3868 (1996).
6. Seth, M. & Ziegler, T. Range-Separated Exchange Functionals with Slater-Type Functions. *J. Chem. Theory Comput.* **8**, 901–907 (2012).
7. Zhao, Y. & Truhlar, D. G. The M06 suite of density functionals for main group thermochemistry, thermochemical kinetics, noncovalent interactions, excited states, and transition elements: two new functionals and systematic testing of four M06-class functionals and 12 other functionals. *Theor Chem Account* **120**, 215–241 (2008).
